# Scenting the Hedonic Connection: Exploring the Impact of Subjective Olfactory Dysfunction on Depressive Symptoms

**DOI:** 10.1002/pchj.828

**Published:** 2025-02-18

**Authors:** Zihan Ni, Ye Liu, Laiquan Zou, Qidong Zhang, Wu Fan, Chao Yan

**Affiliations:** ^1^ Zhengzhou Tobacco Research Institute of CNTC Zhengzhou China; ^2^ School of Psychology and Cognitive Science East China Normal University Shanghai China; ^3^ Chemical Senses and Mental Health Lab, Department of Psychology, School of Public Health Southern Medical University Guangzhou China; ^4^ Shanghai Changning Mental Health Centre Shanghai China; ^5^ Key Laboratory of Philosophy and Social Science of Anhui Province on Adolescent Mental Health and Crisis Intelligence Intervention Hefei China

**Keywords:** chemosensory pleasure, depressive symptoms, hedonic capacity, olfactory function

## Abstract

Olfaction, often regarded as a unique chemical sensation, plays a pivotal role in shaping our quality of life and mental well‐being. Numerous studies have highlighted the significant relationship between olfactory function and depressive symptoms. However, the complex mechanisms underlying how olfactory function affects the development of depressive symptoms remain largely unclear. In this study, we investigated the role of hedonic capacity in the link between olfactory function and depressive symptoms. We recruited 1661 young adults, along with an additional 381 participants who had experienced COVID‐19‐related olfactory dysfunction, to complete a series of self‐report questionnaires assessing depressive symptoms, olfactory dysfunction, and hedonic capacity. A subset of 327 participants completed a follow‐up survey 3 months later. Our sequential mediation analyses revealed that olfactory function indirectly influenced depressive symptoms through chemosensory pleasure. Moreover, it impacted pleasure derived from social activities by modulating chemosensory pleasure. Notably, this mediating effect persisted over the 3‐month period and was evident even in participants with hyposmia, highlighting the lasting importance of chemosensory hedonic capacity. These findings suggest that both chemosensory and social hedonic capacities are crucial in the complex relationship between olfactory function and depressive symptoms. This insight not only deepens our understanding of the developmental psychopathology of depression but also offers a new perspective for its prevention.

## 1 Introduction

Depression, a prevalent mood disorder (American Psychiatric Association 2013), affected an estimated 279.6 million individuals (95% UI 251.6–310.3) in 2019 (Ferrari et al. 2022). This condition poses significant risks to both physical and mental health, leading to disability (Roberts et al. 2018), comorbidities with other medical conditions, and disruptions in social interactions (Luppino et al. 2010). Studies have highlighted changes in olfactory function associated with depression onset (Croy et al. 2014; Negoias et al. 2010; Pollatos et al. 2007), sometimes preceding the appearance of depressive symptoms (Kohli et al. 2016). Following the COVID‐19 pandemic, which notably impacts olfactory abilities, global depression rates have surged by 27.6% (Huang and Zhao 2020; Santomauro et al. 2021). These observations underscore the potential role of olfactory dysfunction in depressive disorders, shedding light on the pathogenesis and progression of depression.

Olfaction plays a crucial role in regulating dietary intake, avoiding environmental hazards, and influencing social interactions (Stevenson 2010). Both human and animal studies provide converging evidence suggesting a potential connection between olfactory dysfunction and depressive symptoms (Athanassi et al. 2021; Kim and Bae 2022; Taalman, Wallace, and Milev 2017). While some studies have primarily established a relationship between olfactory dysfunction and depression symptoms, causality remains inconsistently specified (Pause et al. 2003; Pollatos et al. 2007). Investigations involving humans with olfactory disorders indicate a higher likelihood of depressive symptoms, with approximately one‐quarter to one‐third exhibiting high levels of such symptoms. The severity of depression tends to escalate with the severity of the olfactory disorder, and individuals with such disorders commonly exhibit elevated depression scores (Croy et al. 2014). For instance, a comprehensive cohort study established olfactory dysfunction as a robust predictor of the occurrence of major depressive disorder (MDD) in adults aged 40–65 (Hur et al. 2018). In a follow‐up study of patients with olfactory disorders, Sabiniewicz et al. (2022) found that depressive symptoms varied with olfactory functioning. Among healthy older adults, those with olfactory dysfunction were more prone to frequent depressive symptoms at 5 or 10 years of follow‐up (Eliyan et al. 2020). In addition, animal studies have shown that mice with artificially impaired olfactory function exhibit changes in depression‐related transmitters such as dopamine and serotonin, along with depression‐like behavioral manifestations such as passive avoidance learning deficits and pleasure deficits (Almeida et al. 2017; Chen et al. 2014; Wieronska, Papp, and Pilc 2001). Although there is widespread acknowledgment of the link between olfactory dysfunction and depressive symptoms, the potential mechanism underlying this association remains unclear.

Berridge and Kringelbach (2008) proposed a close relationship between olfactory sensation and pleasure, encompassing three hedonic domains: learning, wanting, and liking (Kringelbach 2005). Anhedonia, characterized by a diminished ability to experience pleasure, is a central symptom of MDD and has been linked to olfactory dysfunction (Deems et al. 1991). Recent investigations in both healthy young adults (Castellano et al. 2023) and children/adolescents (Chen et al. 2022) have identified a connection between momentary levels of olfactory and gustatory sensitivity and hedonic/reward responsiveness. In clinical populations with depression, studies have demonstrated a decline in olfactory identification, with a specific correlation observed between the level of anhedonia and olfactory hedonics (Clepce et al. 2010). Consequently, hedonic capacity is likely a pivotal component in understanding the association between olfactory function and depressive symptoms. However, this area remains underexplored in the literature.

Rizvi et al. (2015) categorized hedonic capacity into four dimensions, encompassing pleasure derived from food/drinks (i.e., the chemosensory domain, including olfactory/gustatory experiences), social activities, pastimes/hobbies, and sensory experiences. Notably, pleasure derived from social activity is closely linked to olfaction and its associated pleasure (Berridge and Kringelbach 2008). Studies of brain structure have demonstrated that hedonics are consistently represented across the olfactory system, from primary to secondary regions, while the social dimension of odors may be processed along a distinct pathway involving social and attentional networks (Mantel et al. 2019). Olfaction serves as a reliable medium for social communication in humans, playing a crucial role in social interactions and the maintenance of well‐being (Blomkvist and Hofer 2021; Boesveldt and Parma 2021). For instance, Boesveldt et al. (2017) found a positive correlation between olfactory recognition ability and the social life of older women. Individuals with olfactory dysfunction have reported increased social isolation and social anhedonia (Keller and Malaspina 2013). Social anhedonia, in turn, is associated with the severity of depression (Atherton, Nevels, and Moore 2015; Enneking et al. 2019; Rey, Jouvent, and Dubal 2009). Considering the recognized social implications of olfaction (Stevenson 2010), our study aimed to investigate whether the influence of olfactory function on chemosensory hedonic capacity extends to overall hedonic capacity, particularly in the realm of social activities, and subsequently impacts depressive symptoms.

Furthermore, given the significant impact of the COVID‐19 virus on human olfactory function, leading to a spectrum of dysfunctions ranging from mild to severe hyposmia (i.e., partial loss of smell), phantosmia (i.e., olfactory hallucinations perceived without external stimuli), and parosmia (i.e., distorted smell perception in the presence of an odorant) (Ciurleo et al. 2020; Hopkins et al. 2021; İşlek and Balcı 2022; Vaira et al. 2020), our objective is to further validate the influence of olfactory hypofunction on both hedonic capacity and depressive symptoms.

We posited the following hypotheses: (1) reduced olfactory function would positively predict depressive symptoms through a sequential mediation pathway involving chemosensory hedonic capacity and general hedonic capacity, with a particular emphasis on social activities; (2) The sequential mediating effect of chemosensory hedonic capacity and social hedonic capacity would remain robust over 3 months; and (3) among individuals experiencing hyposmia, a link between olfactory function and depressive symptoms would persist via a sequential mediation pathway involving chemosensory hedonic capacity and social hedonic capacity.

## 2 Materials and Methods

### 2.1 Participants

Utilizing a freely available application developed by scholars in the R statistical computing language, we conducted a Monte Carlo power analysis employing a method of continuous sample size variation (Schoemann, Boulton, and Short 2017). This analysis was grounded in prior research concerning the correlation coefficients and standard deviations of variables relevant to our study. Specifically, studies reported an association of approximately 0.53 between depression and olfactory dysfunction (Liu et al. 2022), approximately −0.28 between chemosensory pleasurable experiences and depression (Li et al. 2024), and approximately 0.39 between multidimensional anhedonia and depression (Naderi Rajeh et al. 2022). The relationships among olfactory dysfunction, chemosensory pleasure experiences, and multidimensional anhedonia were modeled using Cohen (1977) path coefficient of 0.3, indicative of a medium effect size. To ensure a minimum of 80% statistical power to detect the hypothesized indirect effect, a sample size of approximately 165 participants was calculated as necessary. We recruited 2236 participants using an online crowdsourcing platform (i.e., Sojump) in Chinese mainland, equivalent to Amazon Mechanical Turk. Of those, 1835 were uninfected samples, and 401 samples with a history of COVID‐19 infection. Following the screening process, 174 uninfected samples and 20 COVID‐19‐infected samples were excluded due to incorrect responses to trap questions and the possibility of identical IP addresses and phone numbers, which may have been generated by AI or robots.

As a result, the final uninfected sample comprised 1661 participants (801 males; mean age = 22 ± 2 years). Three months later, 327 uninfected participants (152 males; mean age = 22 ± 2 years) voluntarily continued the study and completed the follow‐up assessment. Additionally, 381 individuals with a prior history of COVID‐19 infection, testing negative for nucleic acid, and reporting a loss of sense of smell were included in subsequent analyses (142 males; mean age = 21 ± 3 years). To meet the eligibility criteria, participants had to be at least 18 years old and free of psychiatric or respiratory diseases. Each participant received 15 yuan as compensation. Informed consent was obtained from all participants. Before completing the questionnaire, participants were required to thoroughly read the informed consent form. Only those who selected the “confirm” option to indicate their consent were permitted to proceed with the survey. Participants who chose not to consent were immediately withdrawn from the study, and their participation was terminated. The study and consent approach were approved by the Human Research Protection Committee of East China Normal University (HR2‐0249‐2021).

### 2.2 Assessments

#### 2.2.1 Self‐Reported Olfactory Dysfunction Questionnaire (SODQ)

The SODQ (Liu et al. 2021) was used to evaluate participants' subjective olfactory dysfunctions. This questionnaire consists of 10 items related to common odor perception issues encountered in daily life. Participants rated their responses on a four‐point Likert scale (0 = *completely disagree*, 3 = *completely agree*), yielding a total score ranging from 0 to 30. Higher scores indicated lower olfactory sensitivity and more severe olfactory impairment. In the current study, the Cronbach's *α* coefficient for the SODQ was calculated to be 0.953, indicating strong reliability.

#### 2.2.2 Beck Depression Inventory‐I (BDI‐I)

We employed the Chinese version of the BDI‐I, as developed by Yeung et al. (2002), to measure depressive symptoms. The questionnaire comprises 21 items that assess the frequency of depressive symptoms over the past week. Responses were recorded on a four‐point Likert scale (0 = *not at all*, 3 = always), with higher scores indicating severer depressive symptoms (Wang et al. 2011). The Cronbach's *α* coefficient was 0.959 in this study.

#### 2.2.3 Chemosensory Pleasant Experience Scale (CPS)

The CPS was utilized to gauge individuals' capacity to experience pleasurable olfactory and gustatory sensations (Qiu et al. 2021). This scale evaluates the extent to which an individual derives pleasure from olfactory and gustatory stimuli. It consists of 12 items across three dimensions—food, imagination, and nature—rated on a 1–6 scale with six levels. Higher scores indicate a heightened ability to derive pleasure from olfactory and gustatory stimuli. In the present study, the Cronbach's *α* coefficient was 0.901.

#### 2.2.4 Dimensional Anhedonia Rating Scale (DARS)

The extent of individual general hedonic capacity was assessed using the Chinese version of DARS (Sheng et al. 2018). This scale comprises two sections containing 17 items, covering four dimensions such as Pastimes/Hobbies (PH), Sensory Experiences (SE), and Social Activities (SA)—are scored on a 5‐point scale (0–4). The aggregate scores, derived from summing the scores of PH, SE, and SA, with higher totals indicating elevated levels of anhedonia. In this study, the Cronbach's *α* coefficient was 0.906.

### 2.3 Statistical Analyses

All statistical analyses were conducted using SPSS 23.0. Pearson correlations were calculated among the core variables, including SODQ score, BDI‐I score, CPS score, and SA/PH/SE score. To explore the sequential mediation model in both the unaffected sample (*N* = 1661) and a sample with a history of COVID‐19 infection (*N* = 381), we utilized Model 6 of the SPSS process plug‐in. The sequential mediating effects of overall hedonic capacity (including hobbies, social activities, and sensory experiences) were assessed independently. Bootstrapping with 5000 samples was utilized to determine statistical significance and produce bias‐corrected 95% confidence intervals (CIs) using the described approach suggested by Preacher and Hayes (2008).

In analyzing longitudinal data, Pearson correlation was employed to examine the associations among olfactory function, chemosensory hedonic capacity at Wave 1, the three general types of hedonic capacity, and depressive symptoms at Wave 2. Following this, a longitudinal sequential mediation model was developed between the two time points.

## 3 Results

### 3.1 Descriptive Characteristics and Correlations Analysis

For the unaffected sample (*N* = 1661), bivariate correlations were conducted using Pearson correlation analysis to examine the relationships among the relevant variables. As shown in Table 1, the results revealed a positive correlation between olfactory dysfunction and depressive symptoms, alongside a negative correlation with chemosensory hedonic capacity, as well as hedonic capacity within the realms of social activities, hobbies, and sensory experiences (*r* = −0.33 to 0.58, *p* < 0.01). Additionally, depressive symptoms exhibited a negative correlation with chemosensory hedonic capacity, hedonic capacity in social activities, hobbies, and sensory experiences (*r* = −0.37 to −0.27, *p* < 0.01). Furthermore, chemosensory hedonic capacity and hedonic capacity in social activities, hobbies, and sensory experiences demonstrated positive intercorrelations (*r* = 0.47 to 0.56, *p* < 0.01). In the sample with a history of COVID‐19 infection (*N* = 381), similar correlations were observed between olfactory dysfunction, hedonic capacity, and depressive symptoms as in uninfected samples (*r* = −0.25 to 0.41, *p* < 0.05) (Table 1).

**TABLE 1:** Correlations between the scale scores in uninfected sample (*N* = 1661) and infected sample (*N* = 381).

	Mean	SD	SODQ	BDI	CPS	DARS_SA	DARS_SE	DARS_PH
General sample without olfactory impairments and COVID‐19 infection (*N* = 1661)								
SODQ	4.530	6.639	—					
BDI	9.798	11.545	0.575**	—				
CPS	59.583	8.218	−0.329**	−0.373**	—			
DARS_SA	13.113	2.511	−0.195**	−0.330**	0.521**	—		
DARS_ SE^a^	16.520	2.940	−0.222**	−0.294**	0.555**	0.689**	—	
DARS_PH	13.475	2.355	−0.246**	−0.273**	0.473**	0.621**	0.652**	—
Sample with COVID‐19 infection and olfactory impairments (*N* = 381)								
SODQ	10.440	9.018	—					
BDI	17.480	16.960	0.373**	—				
CPS	59.680	9.366	−0.213**	−0.244**	—			
DARS_PH	9.460	2.321	−0.211**	−0.186**	0.405**	—		
DARS_SA	12.510	2.839	−0.124*	−0.241**	0.372**	0.342**	—	
DARS_SE^b^	15.710	3.887	−0.195**	−0.250**	0.395**	0.476**	0.423**	—

*Note:* For the DARS_SE, responses related to smell and taste experiences were excluded to prevent potential data overlap. **p < 0.01.

Abbreviations: BDI = Beck Depression Inventory; CPS = Chemosensory Pleasure Scale; DARS_PH = Pastimes/Hobbies in Dimensional Anhedonia Rating Scale; DARS_SA = Social Activities in Dimensional Anhedonia Rating Scale; DARS_SE = Sensory Experience in Dimensional Anhedonia Rating Scale; SODQ = Self‐reported Olfactory Disorder Questionnaire.

^a^ The sample size is 1200.

^b^ The sample size is 309.

In the follow‐up test (*N* = 327), as shown in Table 2, positive correlations emerged between olfactory dysfunction at Wave 1 and depressive symptoms at Wave 2. Conversely, negative correlations were found with chemosensory hedonic capacity at Wave 1 and hedonic capacity in hobbies at Wave 2 (*r* = −0.27 to 0.45, *p* < 0.01). Additionally, similar correlations were observed between chemosensory hedonic capacity at Wave 1, hedonic capacity in social activities/hobbies/sensory experiences and depressive symptoms at Wave 2 (*r* = −0.34 to 0.64, *p* < 0.01).

**TABLE 2:** Correlations between variables in uninfected sample (Wave 1) and follow‐up sample (Wave 2) (*n* = 327).

	*M*	SD	SODQ_W1	CPS_W1	DARS_SA_W2	DARS_SE_W2^a^	DARS_PH_W2	BDI_W2
SODQ_W1	4.645	6.534	—					
CPS_W1	60.037	8.345	−0.271**	—				
DARS_SA_W2	12.991	2.569	−0.080	0.357**	—			
DARS_SE_W2^a^	16.532	2.750	−0.035	0.371**	0.551**	—		
DARS_PH_W2	13.401	2.281	−0.166**	0.403**	0.576**	0.636**	—	
BDI_W2	9.682	10.694	0.452**	−0.324**	−0.325**	−0.211**	0.343**	—

*Note:* For the DARS_SE, responses related to smell and taste experiences were excluded to prevent potential data overlap. **p < 0.01.

Abbreviations: BDI_W2 = Beck Depression Inventory in Wave 2; CPS_W1 = Chemosensory Pleasure Scale in Wave 1; DARS_PH_W2 = Pastimes/Hobbies in Dimensional Anhedonia Rating Scale in Wave 2; DARS_SA_W2 = Social Activities in Dimensional Anhedonia Rating Scale in Wave 2; DARS_SE_W2 = Sensory Experience in Dimensional Anhedonia Rating Scale in Wave 2; SODQ_W1 = Self‐reported Olfactory Disorder Questionnaire in Wave 1.

^a^ The sample size is 220.

### 3.2 Sequential Mediation of Hedonic Capacity

**FIGURE 1:**
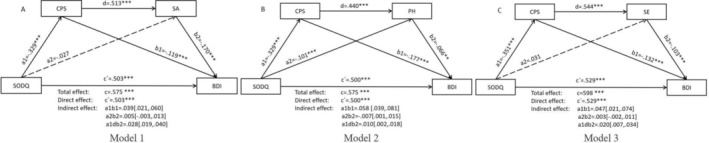
Sequential mediation effect of chemosensory pleasure and general pleasure on the relationship between olfactory dysfunction and depressive symptoms in cross‐sectional data (*N* = 1661). (A) The chain mediating effect of CPS score and SA score on the chain between olfactory dysfunction and depressive symptoms (Model 1); (B) The chain mediating effect of CPS score and PH score on the chain between olfactory dysfunction and depressive symptoms (Model 2). (C) The chain mediating effect of CPS score and SE score on the chain between olfactory dysfunction and depressive symptoms (Model 3). Longitudinal Mediating Modelling. *p < 0.05, ***p < 0.001. BDI = Beck Depression Inventory; CPS = Chemosensory Pleasure Scale; DARS_PH = Pastimes/Hobbies in Dimensional Anhedonia Rating Scale; DARS_SA = Social Activities in Dimensional Anhedonia Rating Scale; DARS_SE = Sensory Experience in Dimensional Anhedonia Rating Scale; SODQ = Self‐reported Olfactory Disorder Questionnaire.

We examined whether the CPS and SA/PH/SE scores could act as sequential mediators in the link between olfactory dysfunction and depressive symptoms. Initially, we observed a significant indirect mediating effect of the CPS score on the positive correlation between SODQ and BDI scores in Model 1 (*B* = 0.039, 95% confidence interval [95% CI] [0.021, 0.060]), Model 2 (*B* = 0.058, 95% CI [0.039, 0.081]), and Model 3 (*B* = 0.047, 95% CI [0.021, 0.074]). Furthermore, sequential mediating analysis results revealed a significant indirect effect of the CPSS score and subsequent SA/PH/SE sub‐scores of the DARS in three distinct models (Model 1: *B* = 0.028, 95% CI [0.019, 0.040]; Model 2: *B* = 0.010, 95% CI [0.002, 0.018]; Model 3: *B* = 0.020, 95% CI [0.007, 0.034]). Specifically, diminished olfactory function led to reduced chemosensory pleasure, subsequently associated with diminished general pleasure derived from social activities, pastimes/hobbies, and sensory experiences, ultimately predicting more severe depressive symptoms. Notably, the direct effect of olfactory dysfunction on depression remained significant in Model 1 (*c* = 0.50, *p* < 0.001), Model 2 (*c* = 0.50, *p* < 0.001), and Model 3 (*c* = 0.53, *p* < 0.001, see Figure 1).

**FIGURE 2:**
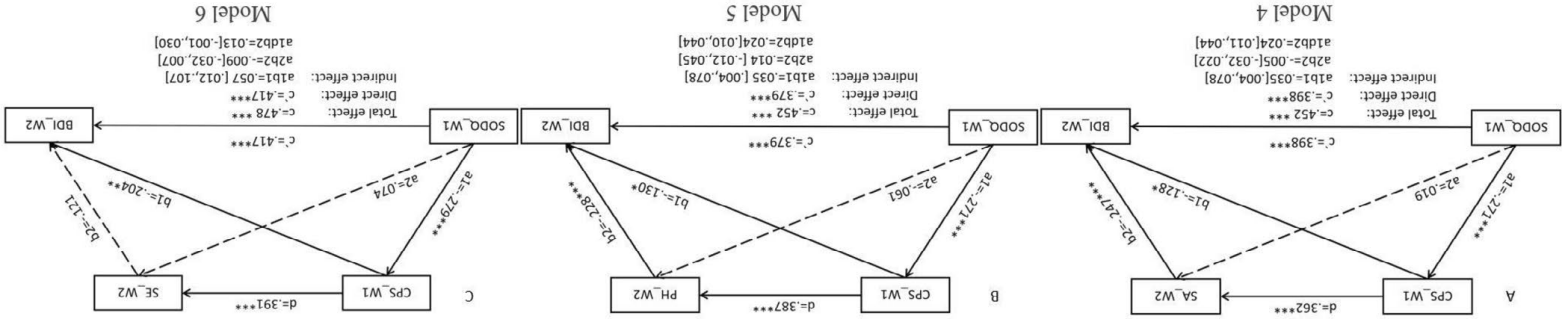
Sequential mediation effect of chemosensory pleasure and general pleasure on the relationship between olfactory dysfunction and depressive symptoms in the longitudinal data (*n* = 327). (A) The chain mediating effect of CPS_W1 score and SA_W2 score on the chain between olfactory dysfunction_W1 and depressive symptoms_W2 (Model 4); (B) The chain mediating effect of CPS_W1 score and PH_W2 score on the chain between olfactory dysfunction_W1 and depressive symptoms_W2 (Model 5). (C) The chain mediating effect of CPS_W1 score and SE_W2 score on the chain between olfactory dysfunction_W1 and depressive symptoms_W2 (Model 6). **p < 0.01, ***p < 0.001. BDI_W2 = Beck Depression Inventory in Wave 2; CPS_W1 = Chemosensory Pleasure Scale in Wave 1; DARS_PH_W2 = Pastimes/Hobbies in Dimensional Anhedonia Rating Scale in Wave 2. DARS_SA_W2 = Social Activities in Dimensional Anhedonia Rating Scale in Wave 2; DARS_SE_W2 = Sensory Experience in Dimensional Anhedonia Rating Scale in Wave 2; SODQ_W1 = Self‐reported Olfactory Disorder Questionnaire in Wave 1.

Furthermore, we examined three mediating models to explore whether the score on the CPS at Wave 1 (CPS_W1) and the SA, PH, and SE sub‐scores of DARS at Wave 2 (SA_W2, PH_W2, SE_W2) would serve as sequential mediators in the relationship between olfactory dysfunction at Wave 1 and depressive symptoms at Wave 2. Consistent with the cross‐sectional data, the indirect effects of chemosensory pleasure at Wave 1 remained significant in the association between olfactory dysfunction at Wave 1 and depressive symptoms at Wave 2 across all three models (Model 4: *B* = 0.035, 95% CI [0.004, 0.078]; Model 5: *B* = 0.035, 95% CI [0.004, 0.078]; Model 6: *B* = 0.057, 95% CI [0.012, 0.107]). Additionally, the analysis of the chain mediating effect revealed two sequential mediating pathways significantly influencing the BDI score at Wave 1 from the SODQ score at Wave 2 through the CPS score at Wave 1 and the scores of SA (Model 4: *B* = 0.024, 95% CI [0.011, 0.044]) and PE (Model 5: *B* = 0.024, 95% CI [0.010, 0.044]) subscales of DARS at Wave 2 (Figure 2). Specifically, diminished olfactory dysfunction at Wave 1 resulted in reduced chemosensory pleasure, which was associated with a subsequent decline in general pleasure from social activity and pastimes/hobbies, ultimately predicting heightened depressive symptoms at Wave 2.

### 3.3 Mediation Effect in Individuals With a History of Olfactory Impairment

**FIGURE 3:**
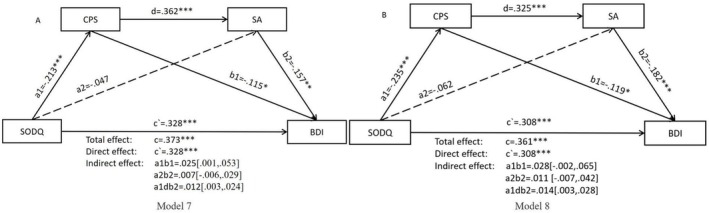
Sequential mediation effect of chemosensory pleasure and hedonic capacity from social activity on the relationship between olfactory dysfunction and depressive symptoms in the sample with olfactory impairments. (A) Mediation model in the samples with a history of olfactory impairments (*n* = 381, Model 7). (B) Mediation model in the samples with a history of olfactory impairments in the hyposmia group (*n* = 320, Model 8) *p < 0.05, **p < 0.01, ***p < 0.001. BDI = Beck Depression Inventory; CPS = Chemosensory Pleasure Scale; DARS_PH = Pastimes/Hobbies in Dimensional Anhedonia Rating Scale; DARS_SA = Social Activities in Dimensional Anhedonia Rating Scale; DARS_SE = Sensory Experience in Dimensional Anhedonia Rating Scale; SODQ = Self‐reported Olfactory Disorder Questionnaire.

In samples with a history of olfactory impairments (*N* = 381), the sequential mediation effect was only significant when the hedonic capacity of SA, a factor of DARS, was utilized as a mediating variable (*B* = 0.012, 95% CI [0.003, 0.024]) (Model 7, Figure 3A). However, when hedonic capacities of PH and SE were considered as mediating variables, the chain mediating effects were not significant. Additionally, we categorized individuals with olfactory disorders into three subtypes: hyposmia, phantosmia, and parosmia. It was observed that only in the hyposmia group, when the hedonic capacity of SA was utilized as a factor of DARS, the sequential mediation effect was significant (*B* = 0.014, 95% CI [0.003, 0.028]) (Model 8, Figure 3B).

## 4 Discussion

This study elucidated the role of hedonic capacities in mediating the impact of olfactory dysfunction on depressive symptoms. In the primary sample, our findings indicated that both chemosensory and socially relevant hedonic capacities sequentially mediated the association between olfactory dysfunction and depressive symptoms. Specifically, olfactory dysfunction either directly led to heightened depressive symptoms by reducing chemosensory pleasure or indirectly influenced depressive symptoms by first diminishing chemosensory pleasure and subsequently affecting the pleasure from social activities. This sequential mediation effect was consistently observed in the 3‐month follow‐up dataset. Furthermore, in the context of COVID‐19‐induced olfactory dysfunction, particularly hyposmia, a similar pattern emerged, with increased depressive symptoms persisting through the sequential mediation of chemosensory and social hedonic capacity.

The findings on the mediating role of chemosensory pleasure are consistent with prior research linking olfactory dysfunction to anhedonia (Castellano et al. 2023; Chen et al. 2022). Since olfactory pleasure is fundamental to hedonic capacity (Kringelbach 2005), a decline in olfactory pleasantness due to reduced olfactory function (Chen et al. 2022), may extend to a broader range of pleasurable feelings, thus impacting depressive symptoms (Castellano et al. 2023; Clepce et al. 2010). At the neuroimaging level, the olfactory pathway intersects with hedonic circuits in key brain regions such as the amygdala, hippocampus, and orbitofrontal cortex (Bear, Connors, and Paradiso 2015; Der‐Avakian and Markou 2012; Yang, Zhu, and Xie 2013). This aligns with the theoretical perspective proposed by Croy et al. (2014), suggesting that decreased olfactory input from malfunction could disrupt the function of the hedonic circuit, potentially influencing the severity of depressive symptoms. Functional magnetic resonance imaging (fMRI) studies employing task‐induced functional connectivity analyses during olfactory and/or trigeminal chemosensory stimulation have shown that individuals with olfactory dysfunction exhibit reduced connections within the hedonic network, including the amygdala, insular cortex, and inferior prefrontal cortex (Kollndorfer et al. 2015). Furthermore, neuroplasticity of the olfactory bulb likely plays a crucial role in hedonic capabilities and depression (Croy and Hummel 2017). A larger olfactory bulb volume is associated with improved olfactory identification and heightened hedonic capacity (Buschhüter et al. 2008; Faria et al. 2024). In individuals with acute MDD, decreased olfactory sensitivity and smaller olfactory bulb volumes have been observed, with the severity of depressive symptoms negatively correlated with olfactory bulb volume and olfactory sensitivity (Negoias et al. 2010). These findings underscore the critical role of chemosensory hedonic capacity in the etiology of depression.

Moreover, our study revealed the sequential mediating roles of chemosensory hedonic capacity and the hedonic capacity of social activities between olfactory function and depression. This effect persisted in the follow‐up data after 3 months, suggesting the prominence and stability of chain mediation. Consistent with our findings, prior research has indicated an inverse correlation between odor identification ability and social anhedonia in individuals with schizotypy (Cieslak et al. 2015; Zou et al. 2015). Considering that olfaction serves as a mechanism for social communication (Stevenson 2010), this result underscores the pivotal role of smell in human social activities (Pause et al. 2004). In group‐living mammals, a phenomenon known as social buffering occurs, where reassuring signals from conspecifics can mitigate stress responses. Social interactions contribute to the downregulation of the hypothalamic–pituitary–adrenal (HPA) axis, offering protective effects against acute and chronic stressors (DeVries, Glasper, and Detillion 2003). Kiyokawa et al. (2009) found that olfactory cues mediate social buffering of stress responses, highlighting the essential role of the olfactory system in this phenomenon. Additionally, Pizzagalli (2014) proposed an integrated model suggesting that anhedonia stems from dysfunctional interactions between stress and reward/hedonic brain circuits, and chronic stressors may elevate the risk of depression by diminishing hedonic capacity. Therefore, social hedonic capacity may also emerge as a potential protective factor against stress, providing valuable insights into the prevention of depressive symptoms.

Interestingly, among the three distinct types of olfactory dysfunction, individuals with hyposmia were the only group demonstrating a significant mediating role for both chemosensory and social hedonic capacity. This further supports the mediating model in the general population and aligns with the prevailing body of research on the link between olfactory dysfunction and depression (Rochet et al. 2018). Following apparent recovery from COVID‐19, some patients reported brain PET hypometabolism in the olfactory gyrus and connected limbic/paralimbic regions (Guedj et al. 2021). In comparison with healthy subjects, hyposmia patients similarly exhibited lower activation in the amygdala, left anterior cingulate, and right OFC when exposed to pleasant odors (Pellegrino et al. 2016). This suggests that declines in olfactory function may influence depressive symptoms through the limbic‐prefrontal pathway (Croy et al. 2014). While Croy, Yarina, and Hummel (2013) found a moderate correlation between phantosmia/parosmia and depressive symptoms in individuals with depression, especially in severe cases where approximately one‐third of patients were suspected or likely to have experienced phantosmia/parosmia, our study did not align with their findings. The lack of significance for phantosmia and parosmia in our current study may be attributed to the relatively small sample size.

Our study has several limitations. First, we assessed olfactory function using a self‐assessment scale, while many previous studies relied on psychophysical odor function tests. This difference may introduce some level of inaccuracy, as self‐reported olfactory loss prevalence is only around 15% (Nordin, Brämerson, and Bende 2004), potentially leading to an overestimation of olfactory function by participants. Future research should consider incorporating both self‐report scales and objective olfactory function evaluation methods, such as Sniffin' Sticks (Haehner et al. 2009), for a more precise assessment. Second, while we validated the reliability and stability of our cross‐sectional findings through a 3‐month follow‐up, this interval may be relatively short compared to the course of depression and long‐term changes in olfactory function, hedonic capacity, and depressive symptoms. Subsequent studies could extend the follow‐up duration, increase sampling time points, and compare the sequential order of interactions between olfactory function, hedonic capacity, and depressive symptoms to better explore causality and influencing mechanisms. Third, it is important to note that our present results cannot imply a causal effect of olfactory function and chemosensory pleasure on the formation of depression. However, in patients with major depression, the introduction of scents has been shown to hasten the reduction of required doses of tricyclic antidepressants (Komori et al. 1995). Consequently, olfactory training, proven effective in enhancing olfactory functioning (Jiramongkolchai et al. 2021; Kollndorfer et al. 2015; Sorokowska et al. 2017), may positively impact depressive symptoms in individuals with subclinical depressive traits (Damm et al. 2014). Moreover, olfactory training has the potential to directly address the causality of olfactory function and chemosensory pleasure in the developmental psychopathology of depression. Beyond the healthy young adult population and individuals with impaired olfactory function following the COVID‐19 pandemic, the relationship between olfactory function, hedonic capacity, and depressive symptoms may be relevant in other groups, such as the elderly, individuals with neurodegenerative diseases, and those with various health conditions (Fatuzzo et al. 2023; Olofsson et al. 2021). In these populations, restoring olfactory function and chemosensory pleasure could play a key role in alleviating depressive symptoms (Bérubé et al. 2023; Pieniak et al. 2022). Therefore, we believe this study offers valuable insights for future mental health intervention strategies.

## 5 Conclusions

Olfactory dysfunction can negatively affect chemosensory hedonic capacity, worsening depressive symptoms. In addition, it can diminish general hedonic capacity, particularly from social activities by reducing the perception of chemosensory pleasure. These findings underscore olfactory dysfunction and its associated diminished hedonic capacity as risk factors for developing depressive symptoms.

## Ethics Statement

This study was approved by the University Committee on Human Research Protection (HR2‐0249‐2021).

## Consent

All persons gave their informed consent prior to their inclusion in the study. Details that might disclose the identity of the subjects under study should be omitted.

## Conflicts of Interest

The authors declare no conflicts of interest.

## Data Availability

The data that support the findings of this study are available on request from the corresponding author. The data are not publicly available due to privacy or ethical restrictions.

## References

[pchj828-bib-0001] Almeida, R. F. d. , M. Ganzella , D. G. Machado , et al. 2017. “Olfactory Bulbectomy in Mice Triggers Transient and Long‐Lasting Behavioral Impairments and Biochemical Hippocampal Disturbances.” Progress in Neuro‐Psychopharmacology and Biological Psychiatry 76: 1–11. 10.1016/j.pnpbp.2017.02.013. 28223107

[pchj828-bib-0002] American Psychiatric Association . 2013. DSM‐5 Task Force, Diagnostic and Statistical Manual of Mental Disorders: DSM‐5™. 5th ed. Washington, DC: American Psychiatric Publishing, Inc. 10.1176/appi.books.9780890425596.

[pchj828-bib-0003] Athanassi, A. , R. Dorado Doncel , K. G. Bath , and N. Mandairon . 2021. “Relationship Between Depression and Olfactory Sensory Function: A Review.” Chemical Senses 46: bjab044. 10.1093/chemse/bjab044. 34618883 PMC8542994

[pchj828-bib-0004] Atherton, B. D. , R. M. Nevels , and M. T. Moore . 2015. “Predicting Symptoms of Depression From Social Anhedonia and Emotion Regulation.” Journal of Nervous and Mental Disease 203, no. 3: 170–174. 10.1097/NMD.0000000000000262. 25668656

[pchj828-bib-0005] Bear, M. F. , B. W. Connors , and M. A. Paradiso . 2015. Neuroscience: Exploring the Brain. 4th ed. Burlington, MA: Jones & Bartlett Learning.

[pchj828-bib-0006] Berridge, K. C. , and M. L. Kringelbach . 2008. “Affective Neuroscience of Pleasure: Reward in Humans and Animals.” Psychopharmacology 199, no. 3: 457–480. 10.1007/s00213-008-1099-6. 18311558 PMC3004012

[pchj828-bib-0007] Bérubé, S. , C. Demers , N. Bussière , et al. 2023. “Olfactory Training Impacts Olfactory Dysfunction Induced by COVID‐19: A Pilot Study.” ORL: Journal for Oto‐rhino‐laryngology and Its Related Specialties 85, no. 2: 57–66. 10.1159/000528188. 36529118 PMC9843729

[pchj828-bib-0008] Blomkvist, A. , and M. Hofer . 2021. “Olfactory Impairment and Close Social Relationships. A Narrative Review.” Chemical Senses 46: bjab037. 10.1093/chemse/bjab037. 34351415 PMC8385889

[pchj828-bib-0009] Boesveldt, S. , and V. Parma . 2021. “The Importance of the Olfactory System in Human Well‐Being, Through Nutrition and Social Behavior.” Cell and Tissue Research 383, no. 1: 559–567. 10.1007/s00441-020-03367-7. 33433688 PMC7802608

[pchj828-bib-0010] Boesveldt, S. , J. R. Yee , M. K. McClintock , and J. N. Lundström . 2017. “Olfactory Function and the Social Lives of Older Adults: A Matter of Sex.” Scientific Reports 7, no. 1: 45118. 10.1038/srep45118. 28327569 PMC5361152

[pchj828-bib-0011] Buschhüter, D. , M. Smitka , S. Puschmann , et al. 2008. “Correlation Between Olfactory Bulb Volume and Olfactory Function.” NeuroImage 42, no. 2: 498–502. 10.1016/j.neuroimage.2008.05.004. 18555701

[pchj828-bib-0012] Castellano, P. , V. Gigli , V. Ghezzi , et al. 2023. “Momentary Gustative‐Olfactory Sensitivity and Tonic Heart Rate Variability Are Independently Associated With Motivational Behavior.” International Journal of Psychophysiology 186: 1–9. 10.1016/j.ijpsycho.2023.01.010. 36738932

[pchj828-bib-0013] Chen, Y. , X. Liu , X. Jia , et al. 2014. “Anxiety‐ and Depressive‐Like Behaviors in Olfactory Deficient Cnga2 Knockout Mice.” Behavioural Brain Research 275: 219–224. 10.1016/j.bbr.2014.08.042. 25192635

[pchj828-bib-0014] Chen, Y. , Y. Zhan , Y. Qiu , J. Zhao , and L. Zou . 2022. “Odor Identification Ability as a Mediator of Schizotypal Traits and Odor Hedonic Capacity in Non‐Clinical Children and Adolescents.” Brain Sciences 12, no. 5: 534. https://www.mdpi.com/2076‐3425/12/5/534. 35624921 10.3390/brainsci12050534 PMC9138986

[pchj828-bib-0015] Cieslak, K. , J. Walsh‐Messinger , A. Stanford , et al. 2015. “Olfactory Performance Segregates Effects of Anhedonia and Anxiety on Social Function in Patients With Schizophrenia.” Journal of Psychiatry & Neuroscience 40, no. 6: 387–393. 10.1503/jpn.140268. 26107162 PMC4622636

[pchj828-bib-0016] Ciurleo, R. , S. De Salvo , L. Bonanno , S. Marino , P. Bramanti , and F. Caminiti . 2020. “Parosmia and Neurological Disorders: A Neglected Association.” Frontiers in Neurology 11: 543275. 10.3389/fneur.2020.543275. 33240192 PMC7681001

[pchj828-bib-0017] Clepce, M. , A. Gossler , K. Reich , J. Kornhuber , and N. Thuerauf . 2010. “The Relation Between Depression, Anhedonia and Olfactory Hedonic Estimates—A Pilot Study in Major Depression.” Neuroscience Letters 471, no. 3: 139–143. 10.1016/j.neulet.2010.01.027. 20097263

[pchj828-bib-0018] Cohen, J. 1977. “Chapter 7—Chi‐Square Tests for Goodness of Fit and Contingency Tables.” In Statistical Power Analysis for the Behavioral Sciences, edited by J. Cohen , 215–271. New York: Academic Press. 10.1016/B978-0-12-179060-8.50012-8.

[pchj828-bib-0019] Croy, I. , and T. Hummel . 2017. “Olfaction as a Marker for Depression.” Journal of Neurology 264, no. 4: 631–638. 10.1007/s00415-016-8227-8. 27393116

[pchj828-bib-0020] Croy, I. , A. Symmank , J. Schellong , et al. 2014. “Olfaction as a Marker for Depression in Humans.” Journal of Affective Disorders 160: 80–86. 10.1016/j.jad.2013.12.026. 24445134

[pchj828-bib-0021] Croy, I. , S. Yarina , and T. Hummel . 2013. “Research Letter Enhanced Parosmia and Phantosmia in Patients With Severe Depression.” Psychological Medicine 43, no. 11: 2460–2464. 10.1017/S0033291713001773. 23883685

[pchj828-bib-0022] Damm, M. , L. K. Pikart , H. Reimann , et al. 2014. “Olfactory Training Is Helpful in Postinfectious Olfactory Loss: A Randomized, Controlled, Multicenter Study.” Laryngoscope 124, no. 4: 826–831. 10.1002/lary.24340. 23929687

[pchj828-bib-0023] Deems, D. A. , R. L. Doty , R. G. Settle , et al. 1991. “Smell and Taste Disorders, a Study of 750 Patients From the University of Pennsylvania Smell and Taste Center.” Archives of Otolaryngology—Head & Neck Surgery 117, no. 5: 519–528. 10.1001/archotol.1991.01870170065015. 2021470

[pchj828-bib-0024] Der‐Avakian, A. , and A. Markou . 2012. “The Neurobiology of Anhedonia and Other Reward‐Related Deficits.” Trends in Neurosciences 35, no. 1: 68–77. 10.1016/j.tins.2011.11.005. 22177980 PMC3253139

[pchj828-bib-0025] DeVries, A. C. , E. R. Glasper , and C. E. Detillion . 2003. “Social Modulation of Stress Responses.” Physiology & Behavior 79, no. 3: 399–407. 10.1016/S0031-9384(03)00152-5. 12954434

[pchj828-bib-0026] Eliyan, Y. , K. E. Wroblewski , M. K. McClintock , and J. M. Pinto . 2020. “Olfactory Dysfunction Predicts the Development of Depression in Older US Adults.” Chemical Senses 46: bjaa075. 10.1093/chemse/bjaa075. PMC7909297 33197253

[pchj828-bib-0027] Enneking, V. , P. Krüssel , D. Zaremba , et al. 2019. “Social Anhedonia in Major Depressive Disorder: A Symptom‐Specific Neuroimaging Approach.” Neuropsychopharmacology 44, no. 5: 883–889. 10.1038/s41386-018-0283-6. 30607014 PMC6461766

[pchj828-bib-0028] Faria, V. , A. Joshi , C. Mignot , D. Thaploo , S. Weise , and T. Hummel . 2024. “Neuroimaging the Development of Olfactory Function in a Woman With no Olfactory Bulbs.” JAMA Otolaryngology: Head & Neck Surgery 150, no. 1: 81. 10.1001/jamaoto.2023.3667. 38032618

[pchj828-bib-0029] Fatuzzo, I. , G. F. Niccolini , F. Zoccali , et al. 2023. “Neurons, Nose, and Neurodegenerative Diseases: Olfactory Function and Cognitive Impairment.” International Journal of Molecular Sciences 24, no. 3: 2117. https://www.mdpi.com/1422‐0067/24/3/2117. 36768440 10.3390/ijms24032117 PMC9916823

[pchj828-bib-0030] Ferrari, A. J. , D. F. Santomauro , A. M. M. Herrera , et al. 2022. “Global, Regional, and National Burden of 12 Mental Disorders in 204 Countries and Territories, 1990–2019: A Systematic Analysis for the Global Burden of Disease Study 2019.” Lancet Psychiatry 9, no. 2: 137–150. 10.1016/S2215-0366(21)00395-3. 35026139 PMC8776563

[pchj828-bib-0031] Guedj, E. , J. Y. Campion , P. Dudouet , et al. 2021. “18F‐FDG Brain PET Hypometabolism in Patients With Long COVID.” European Journal of Nuclear Medicine and Molecular Imaging 48, no. 9: 2823–2833. 10.1007/s00259-021-05215-4. 33501506 PMC7837643

[pchj828-bib-0032] Haehner, A. , A. M. Mayer , B. N. Landis , et al. 2009. “High Test–Retest Reliability of the Extended Version of the “Sniffin’ Sticks” Test.” Chemical Senses 34, no. 8: 705–711. 10.1093/chemse/bjp057. 19759361

[pchj828-bib-0033] Hopkins, C. , P. Surda , L. A. Vaira , et al. 2021. “Six Month Follow‐Up of Self‐Reported Loss of Smell During the COVID‐19 Pandemic.” Rhinology 59, no. 1: 26. 10.4193/Rhin20.544. 33320115

[pchj828-bib-0034] Huang, Y. , and N. Zhao . 2020. “Generalized Anxiety Disorder, Depressive Symptoms and Sleep Quality During COVID‐19 Outbreak in China: A Web‐Based Cross‐Sectional Survey.” Psychiatry Research 288: 112954. 10.1016/j.psychres.2020.112954. 32325383 PMC7152913

[pchj828-bib-0035] Hur, K. , J. S. Choi , M. Zheng , J. Shen , and B. Wrobel . 2018. “Association of Alterations in Smell and Taste With Depression in Older Adults.” Laryngoscope Investigative Otolaryngology 3, no. 2: 94–99. 10.1002/lio2.142. 29721540 PMC5915822

[pchj828-bib-0036] İşlek, A. , and M. K. Balcı . 2022. “Phantosmia With COVID‐19 Related Olfactory Dysfunction: Report of Nine Case.” Indian Journal of Otolaryngology and Head & Neck Surgery 74, no. 2: 2891–2893. 10.1007/s12070-021-02505-z. 33728275 PMC7953190

[pchj828-bib-0037] Jiramongkolchai, P. , M. S. Jones , A. Peterson , et al. 2021. “Association of Olfactory Training With Neural Connectivity in Adults With Postviral Olfactory Dysfunction.” JAMA Otolaryngology: Head & Neck Surgery 147, no. 6: 502–509. 10.1001/jamaoto.2021.0086. 33734298 PMC7974830

[pchj828-bib-0038] Keller, A. , and D. Malaspina . 2013. “Hidden Consequences of Olfactory Dysfunction: A Patient Report Series.” BMC Ear, Nose and Throat Disorders 13, no. 1: 8. 10.1186/1472-6815-13-8. 23875929 PMC3733708

[pchj828-bib-0039] Kim, B.‐Y. , and J. H. Bae . 2022. “Olfactory Function and Depression: A Meta‐Analysis.” Ear, Nose & Throat Journal 104, no. 1: 39–46. 10.1177/01455613211056553. 35360974

[pchj828-bib-0040] Kiyokawa, Y. , Y. Takeuchi , M. Nishihara , and Y. Mori . 2009. “Main Olfactory System Mediates Social Buffering of Conditioned Fear Responses in Male Rats.” European Journal of Neuroscience 29, no. 4: 777–785. 10.1111/j.1460-9568.2009.06618.x. 19250440

[pchj828-bib-0041] Kohli, P. , Z. M. Soler , S. A. Nguyen , J. S. Muus , and R. J. Schlosser . 2016. “The Association Between Olfaction and Depression: A Systematic Review.” Chemical Senses 41, no. 6: 479–486. 10.1093/chemse/bjw061. 27170667 PMC4918728

[pchj828-bib-0042] Kollndorfer, K. , F. P. S. Fischmeister , K. Kowalczyk , et al. 2015. “Olfactory Training Induces Changes in Regional Functional Connectivity in Patients With Long‐Term Smell Loss.” NeuroImage Clinical 9: 401–410. 10.1016/j.nicl.2015.09.004. 26594622 PMC4590718

[pchj828-bib-0043] Komori, T. , R. Fujiwara , M. Tanida , J. Nomura , and M. M. Yokoyama . 1995. “Effects of Citrus Fragrance on Immune Function and Depressive States.” Neuroimmunomodulation 2, no. 3: 174–180. 10.1159/000096889. 8646568

[pchj828-bib-0044] Kringelbach, M. L. 2005. “The Human Orbitofrontal Cortex: Linking Reward to Hedonic Experience.” Nature Reviews Neuroscience 6, no. 9: 691–702. 10.1038/nrn1747. 16136173

[pchj828-bib-0045] Li, J. , B. Chen , Q. Wang , et al. 2024. “Chemosensory Anhedonia Facilitates Depressive Symptoms and Cognitive Impairment in Late‐Life Depression.” Geriatrics & Gerontology International 24, no. 10: 1022–1029. 10.1111/ggi.14968. 39266228

[pchj828-bib-0046] Liu, D. T. , B. Prem , G. Sharma , J. Kaiser , G. Besser , and C. A. Mueller . 2022. “Depression Symptoms and Olfactory‐Related Quality of Life.” Laryngoscope 132, no. 9: 1829–1834. 10.1002/lary.30122. 35353380 PMC9544892

[pchj828-bib-0047] Liu, X. , J. Huang , P. Tian , J. Hu , and L. Zou . 2021. “Development of a Self‐Reported Olfactory Dysfunction Questionnaire (SODQ) to Screen Olfactory Disorders in China.” Rhinology 59, no. 4: 393–397. 10.4193/Rhin21.028. 34129661

[pchj828-bib-0048] Luppino, F. S. , L. M. de Wit , P. F. Bouvy , et al. 2010. “Overweight, Obesity, and Depression: A Systematic Review and Meta‐Analysis of Longitudinal Studies.” Archives of General Psychiatry 67, no. 3: 220–229. 10.1001/archgenpsychiatry.2010.2. 20194822

[pchj828-bib-0049] Mantel, M. , C. Ferdenzi , J.‐M. Roy , and M. Bensafi . 2019. “Individual Differences as a Key Factor to Uncover the Neural Underpinnings of Hedonic and Social Functions of Human Olfaction: Current Findings From PET and fMRI Studies and Future Considerations.” Brain Topography 32, no. 6: 977–986. 10.1007/s10548-019-00733-9. 31564029

[pchj828-bib-0050] Naderi Rajeh, Y. , B. Dolatshahi , A. Pourshahbaz , and M. Zarghami . 2022. “Assessing the Validity and Reliability of the Dimensional Anhedonia Rating Scale (DARS) for the Iranian Population.” International Journal of Psychiatry and Behavioral Sciences 16, no. 2: e120043. 10.5812/ijpbs-120043.

[pchj828-bib-0051] Negoias, S. , I. Croy , J. Gerber , et al. 2010. “Reduced Olfactory Bulb Volume and Olfactory Sensitivity in Patients With Acute Major Depression.” Neuroscience 169, no. 1: 415–421. 10.1016/j.neuroscience.2010.05.012. 20472036

[pchj828-bib-0053] Nordin, S. , A. Brämerson , and M. Bende . 2004. “Prevalence of Self‐Reported Poor Odor Detection Sensitivity: The Skövde Population‐Based Study.” Acta Oto‐Laryngologica 124, no. 10: 1171–1173. 10.1080/00016480410017468. 15768812

[pchj828-bib-0054] Olofsson, J. K. , I. Ekström , M. Larsson , and S. Nordin . 2021. “Olfaction and Aging: A Review of the Current State of Research and Future Directions.” Perception 12, no. 3: 20416695211020331. 10.1177/20416695211020331. PMC8239976 34249327

[pchj828-bib-0055] Pause, B. M. , A. Ohrt , A. Prehn , and R. Ferstl . 2004. “Positive Emotional Priming of Facial Affect Perception in Females Is Diminished by Chemosensory Anxiety Signals.” Chemical Senses 29, no. 9: 797–805. 10.1093/chemse/bjh245. 15574815

[pchj828-bib-0056] Pause, B. M. , N. Raack , B. Sojka , R. Göder , J. B. Aldenhoff , and R. Ferstl . 2003. “Convergent and Divergent Effects of Odors and Emotions in Depression.” Psychophysiology 40, no. 2: 209–225. 10.1111/1469-8986.00023. 12820862

[pchj828-bib-0057] Pellegrino, R. , A. Hähner , V. Bojanowski , C. Hummel , J. Gerber , and T. Hummel . 2016. “Olfactory Function in Patients With Hyposmia Compared to Healthy Subjects—An fMRI Study.” Rhinology 54, no. 4: 374–381. 10.4193/Rhino16.098. 27421303

[pchj828-bib-0058] Pieniak, M. , A. Oleszkiewicz , V. Avaro , F. Calegari , and T. Hummel . 2022. “Olfactory Training—Thirteen Years of Research Reviewed.” Neuroscience & Biobehavioral Reviews 141: 104853. 10.1016/j.neubiorev.2022.104853. 36064146

[pchj828-bib-0059] Pizzagalli, D. A. 2014. “Depression, Stress, and Anhedonia: Toward a Synthesis and Integrated Model.” Annual Review of Clinical Psychology 10, no. 1: 393–423. 10.1146/annurev-clinpsy-050212-185606. PMC3972338 24471371

[pchj828-bib-0060] Pollatos, O. , J. Albrecht , R. Kopietz , et al. 2007. “Reduced Olfactory Sensitivity in Subjects With Depressive Symptoms.” Journal of Affective Disorders 102, no. 1: 101–108. 10.1016/j.jad.2006.12.012. 17291590

[pchj828-bib-0061] Preacher, K. J. , and A. F. Hayes . 2008. “Asymptotic and Resampling Strategies for Assessing and Comparing Indirect Effects in Multiple Mediator Models.” Behavior Research Methods 40, no. 3: 879–891. 10.3758/brm.40.3.879. 18697684

[pchj828-bib-0062] Qiu, Y.‐q. , G.‐j. Huang , J.‐b. Zhao , Q.‐w. Ma , and L.‐q. Zou . 2021. “The Chemosensory Pleasure Scale for Children (CPS‐C): Factor Structure, Reliability, and Validity.” Food Quality and Preference 92: 104214. 10.1016/j.foodqual.2021.104214.

[pchj828-bib-0063] Rey, G. , R. Jouvent , and S. Dubal . 2009. “Schizotypy, Depression, and Anxiety in Physical and Social Anhedonia.” Journal of Clinical Psychology 65, no. 7: 695–708. 10.1002/jclp.20577. 19388058

[pchj828-bib-0064] Rizvi, S. J. , A. Zaretsky , A. Schaffer , and A. Levitt . 2015. “Is Immediate Adjunctive CBT More Beneficial Than Delayed CBT in Treating Depression?: A Pilot Study.” Journal of Psychiatric Practice 21, no. 2: 107–113. 10.1097/01.pra.0000462603.71983.15. 25782761

[pchj828-bib-0065] Roberts, N. L. S. , W. C. Mountjoy‐Venning , M. Anjomshoa , J. A. M. Banoub , and Y. J. Yasin . 2018. “Global, Regional, and National Incidence, Prevalence, and Years Lived With Disability for 354 Diseases and Injuries for 195 Countries and Territories, 1990–2017: A Systematic Analysis for the Global Burden of Disease Study 2017.” Lancet 392, no. 10159: 1789–1858. 10.1016/s0140-6736(18)32279-7. 30496104 PMC6227754

[pchj828-bib-0066] Rochet, M. , W. El‐Hage , S. Richa , F. Kazour , and B. Atanasova . 2018. “Depression, Olfaction, and Quality of Life: A Mutual Relationship.” Brain Sciences 8, no. 5: 80. https://www.mdpi.com/2076‐3425/8/5/80. 29734670 10.3390/brainsci8050080 PMC5977071

[pchj828-bib-0067] Sabiniewicz, A. , L. Hoffmann , A. Haehner , and T. Hummel . 2022. “Symptoms of Depression Change With Olfactory Function.” Scientific Reports 12, no. 1: 5656. 10.1038/s41598-022-09650-7. 35383250 PMC8983665

[pchj828-bib-0068] Santomauro, D. F. , A. M. Mantilla Herrera , J. Shadid , et al. 2021. “Global Prevalence and Burden of Depressive and Anxiety Disorders in 204 Countries and Territories in 2020 due to the COVID‐19 Pandemic.” Lancet 398, no. 10312: 1700–1712. 10.1016/S0140-6736(21)02143-7. 34634250 PMC8500697

[pchj828-bib-0069] Schoemann, A. M. , A. J. Boulton , and S. D. Short . 2017. “Determining Power and Sample Size for Simple and Complex Mediation Models.” Social Psychological and Personality Science 8, no. 4: 379–386. 10.1177/1948550617715068.

[pchj828-bib-0070] Sheng, R.‐r. , Q.‐q. Wu , F.‐y. Zhang , B. Shi , F.‐q. Yu , and C.‐y. Zhu . 2018. “Reliability and Validity of Chinese Version of Dimensional Anhedonia Rating Scale Used in Undergraduates.” Modern Preventive Medicine 45, no. 22: 5.

[pchj828-bib-0071] Sorokowska, A. , E. Drechsler , M. Karwowski , and T. Hummel . 2017. “Effects of Olfactory Training: A Meta‐Analysis.” Rhinology 55, no. 1: 17–26. 10.4193/Rhin16.195. 28040824

[pchj828-bib-0072] Stevenson, R. J. 2010. “An Initial Evaluation of the Functions of Human Olfaction.” Chemical Senses 35, no. 1: 3–20. 10.1093/chemse/bjp083. 19942579

[pchj828-bib-0073] Taalman, H. , C. Wallace , and R. Milev . 2017. “Olfactory Functioning and Depression: A Systematic Review.” Frontiers in Psychiatry 8: 190. 10.3389/fpsyt.2017.00190. 29033860 PMC5627007

[pchj828-bib-0074] Vaira, L. A. , C. Hopkins , G. Salzano , et al. 2020. “Olfactory and Gustatory Function Impairment in COVID‐19 Patients: Italian Objective Multicenter‐Study.” Head & Neck 42, no. 7: 1560–1569. 10.1002/hed.26269. 32437022 PMC7280583

[pchj828-bib-0075] Wang, Z. , C.‐M. Yuan , J. Huang , et al. 2011. “Reliability and Validity of the Chinese Version of Beck Depression Inventory‐II Among Depression Patients.” Chinese Mental Health Journal 25, no. 6: 476–480.

[pchj828-bib-0076] Wieronska, J. M. , M. Papp , and A. Pilc . 2001. “Effects of Anxiolytic Drugs on Some Behavioral Consequences in Olfactory Bulbectomized Rats.” Polish Journal of Pharmacology 53, no. 5: 517–525. 11990071

[pchj828-bib-0077] Yang, X.‐h. , C.‐y. Zhu , and G.‐r. Xie . 2013. “Anhedonia in Depression: Definition and Neuralbiological Mechanism.” Chinese Journal of Clinical Psychology 21, no. 5: 747–750. 10.16128/j.cnki.1005-3611.2013.05.043.

[pchj828-bib-0078] Yeung, A. , S. Howarth , R. Chan , S. Sonawalla , A. A. Nierenberg , and M. Fava . 2002. “Use of the Chinese Version of the Beck Depression Invention for Screening Depression in Primary Care.” Journal of Nervous and Mental Disease 190, no. 2: 94–99. 10.1097/00005053-200202000-00005. 11889362

[pchj828-bib-0082] Zou, L.‐q. , F.‐l. Geng , W.‐h. Liu , et al. 2015. “The Neural Basis of Olfactory Function and Its Relationship With Anhedonia in Individuals With Schizotypy: An Exploratory Study.” Psychiatry Research: Neuroimaging 234, no. 2: 202‐207. 10.1016/j.pscychresns.2015.09.011. 26404551

